# Moderators of the effectiveness of an intervention to increase colorectal cancer screening through mailed fecal immunochemical test kits: results from a pragmatic randomized trial

**DOI:** 10.1186/s13063-019-4027-7

**Published:** 2020-01-15

**Authors:** Elizabeth A. O’Connor, William M. Vollmer, Amanda F. Petrik, Beverly B. Green, Gloria D. Coronado

**Affiliations:** 1 Kaiser Permanente Center for Health Research, 3800 N. Interstate Avenue, Portland, OR 97227 USA; 2 Kaiser Permanente Washington Health Research Institute, 1730 Minor Avenue, Suite, Seattle, WA 1600 USA

**Keywords:** Colorectal cancer, Prevention, Screening, Fecal immunochemical test, Disparities

## Abstract

**Background:**

Colorectal cancer (CRC) screening rates remain suboptimal, particularly in low-income and underserved populations. Mailed fecal immunochemical testing (FIT) may overcome common barriers to screening; however, the effect of mailed FIT kits may differ across important subpopulations. The goal of the current study was to examine sociodemographic and health-related factors that moderate the effect of an intervention of automated direct mail of FIT kits at health clinics serving low-income populations.

**Methods:**

This study is a secondary analysis of the Strategies and Opportunities to Stop Colon Cancer in Priority Populations (STOP CRC) study, a cluster-randomized pragmatic trial to increase uptake of CRC screening in patients seen at federally qualified health centers. The intervention involved tools embedded in the electronic medical records to enable participating clinics to mail FIT kits and related materials to eligible participants. We examined the rate of FIT completion by potential moderating characteristics using electronic health record data supplemented by the American Community Survey and the Centers for Medicare & Medicaid Services Geographic Variation datasets, linked via geocoding to patients’ addresses. All patients aged 50–75 seen in participating health clinics who were eligible for CRC screening were included.

**Results:**

Although not always statistically significant, we saw a consistent pattern of increased FIT return rates among intervention participants compared to control participants across all subgroups studied, with incidence rate ratios (IRRs) generally ranging from 1.25 to 1.50. FIT completion in the intervention group ranged from 15 and 20% across subpopulations, typically three to six percentage points higher than the control group participants. The only moderator with a statistically significant interaction was race: persons of Asian descent showed a twofold response to the intervention (adjusted incidence rate ratio [aIRR] = 2.06, 95% confidence interval 1.41 to 3.00).

**Conclusions:**

Response to a mailed FIT intervention was generally consistent across a wide range of individual and neighborhood-level patient characteristics, including typically underserved patients and those in low-resource communities.

**Trial registration:**

ClinicalTrials.gov, NCT01742065. Registered on 5 December 2012.

## Background

Colorectal cancer (CRC) is one of the leading causes of cancer mortality [1, 2]. The US Preventive Services Task Force (USPSTF) gives CRC screening an A-level recommendation for adults aged 50 to 75 [3], and this service is among the highest rated clinical preventive services in the USPSTF’s portfolio for its potential to avoid morbidity and mortality and also save costs [4]. A microsimulation model estimated that annual fecal immunochemical testing (FIT) among adults aged 50 to 75 would result in 244 life-years gained per 1000 persons, and other CRC screening methods (e.g., periodic sigmoidoscopy and colonoscopy) showed similar levels of benefit [5]. Despite this, CRC screening is well below targets set by both Healthy People 2020 [6] and the National Colorectal Cancer Roundtable [7].

In addition, there are disparities in CRC screening rates. According to the National Health Interview Survey, CRC screening rates are lower for those with low income, lack of health insurance, low education levels, who lack a source of regular medical care, or who are recent immigrants [8]. Rates are also lower in several race/ethnicity subgroups, including patients who are Hispanic, Native Hawaiian or other Pacific Islander, and American Indian/Alaska Native [9]. CRC screening is also associated with a number of health-related factors, such as the presence of medical conditions [10–13] and utilization of other preventive health services [10, 12].

CRC screening is typically initiated at a medical visit, but there are important known barriers to this approach, such as cost, lack of health insurance, and difficulty attending medical appointments. A mail-based intervention may boost CRC screening rates and reduce disparities in underserved populations by reducing these barriers. A number of studies have shown that mailing FIT kits directly to patients can substantially increase screening rates in low-income, minority, and racially diverse settings [14–20]. Screening rates were variable in these studies, ranging from 2 to 37% at baseline, and with the introduction of a FIT kit, mailing program rates increased by a factor of two to six, with absolute changes typically ranging from 21 to 29 percentage points. Two trials found that mailing FIT kits to patients who were unscreened was more effective in increasing CRC screening than phoning people to schedule colonoscopy appointments after 1 year [16, 20], although this effect did not hold up with a 3-year follow-up [21, 22]. Further, a recent study in a health maintenance organization (HMO) setting demonstrated that, among patients who had completed one FIT, 75–86% completed two additional rounds of screening within 4 years, suggesting good acceptability of this screening method among those who had used it [23]. Similarly, in a study of veterans who had completed a FIT, 89% found it easy to use and convenient, and 97% reported that they were likely to complete a FIT by mail annually [24]. In this group of veterans, 79% completed a second annual FIT test by mail [24].

Understanding whether mailed FIT interventions are broadly effective could assure health systems administrators that this approach would benefit a wide swath of patients and be unlikely to exacerbate or introduce disparities. This study explores whether sociodemographic and health-related factors moderate the effect of an automated direct mail of FIT kit program delivered to patients receiving care at health clinics serving primarily low-income populations.

## Materials and methods

This study is a secondary analysis of data from the Strategies and Opportunities to Stop Colon Cancer in Priority Populations (STOP CRC) study, a cluster-randomized pragmatic trial to increase uptake of CRC screening [25]. The study was approved by the Institutional Review Board of Kaiser Permanente Northwest (Protocol # 4364), with ceding agreements from Group Health Research Institute and OCHIN (formerly Oregon Community Health Information Network), and is registered at ClinicalTrials.gov (NCT01742065).

### Study design and randomization

**Fig. 1:**
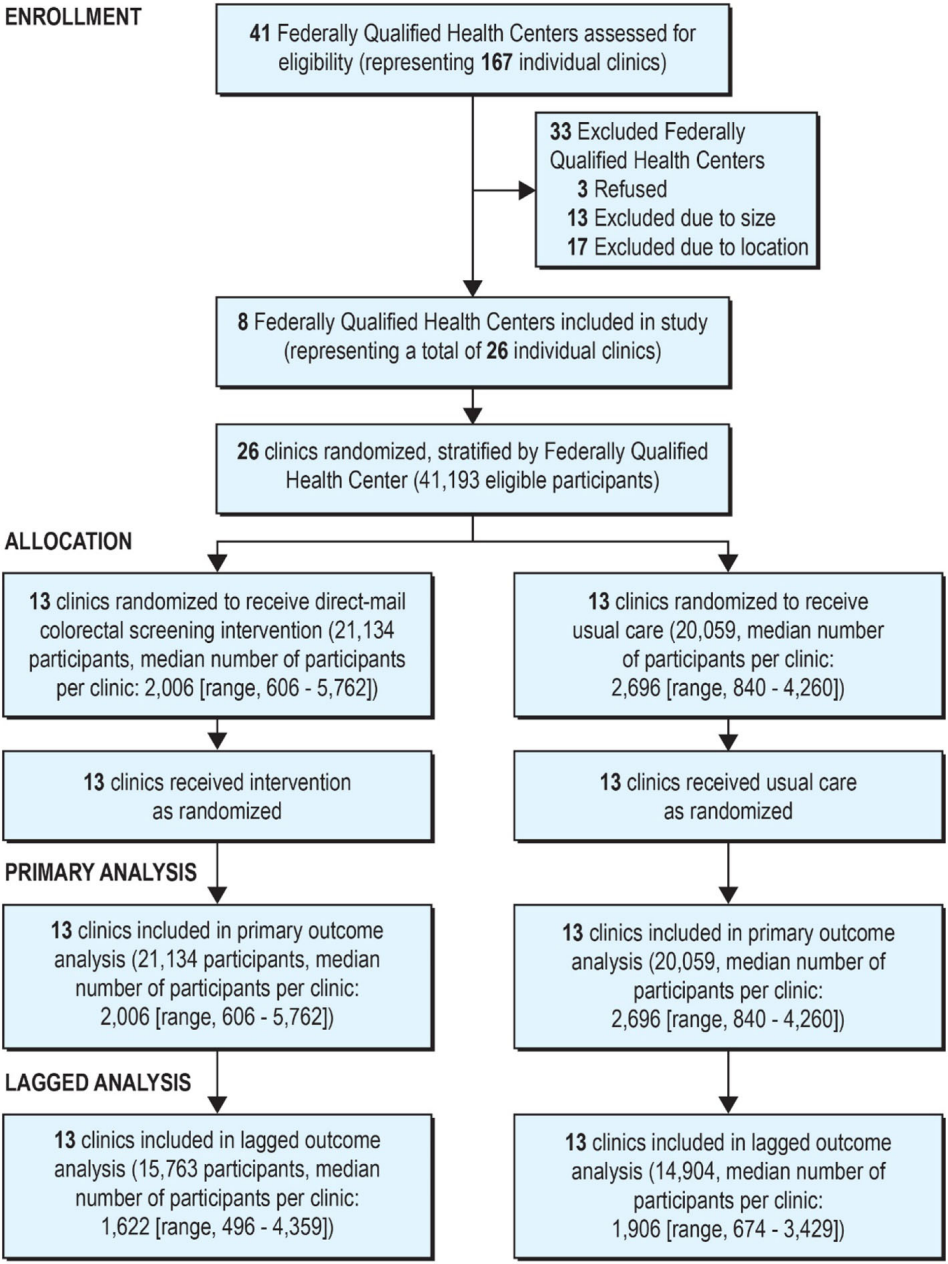
CONSORT Flowchart of the STOP CRC study

The design of the parent trial is described elsewhere in detail [25, 26] and is only summarized here. Primary attention here is focused on methods unique to this secondary analysis. Twenty-six clinics from eight federally qualified health centers serving low-income populations were randomized in a one-to-one ratio using a computer-generated randomization strategy prepared by a statistician. Neither clinic staff nor research staff had access to the allocation schedule prior to randomization. Allocation assignments were stratified by health center and blocked to assure maximum balance within health centers. Clinics were required to have a minimum of 450 patients aged 50–75 years as well as the necessary clinical and laboratory capacity and electronic health record (EHR) infrastructure to comply with the study’s requirements. Randomization occurred in February 2014. Due to startup delays trigged by a scheduled upgrade to the EHR, clinics were unable to begin intervention activities until May 2014. As a result, we developed a secondary, lagged dataset that effectively did not begin recruiting intervention or control participants until May 2014 [27]. Sensitivity analyses using this lagged dataset were conducted for the cohort overall to provide what we believe is a more accurate estimate of the true intervention effect [25]. For similar reasons, and to maximize power to observe subgroup and interaction effects, it is this cohort that was used for the present analysis as well (Fig. 1).

### Participants

Patients from both intervention and control clinics were included in the primary analysis sample if, at any time during the first 12 months post randomization, they were aged 50–75 years, did not already have CRC or other exclusionary diagnoses for CRC screening, and were not compliant with current USPSTF guidelines for screening [3]. The date this occurred defined the starting point for follow-up assessment for each individual.

### Intervention

Tools were developed to enable clinics to use the EHR to generate mailing lists and materials for a series of three mailings: (1) an introductory letter, (2) a FIT kit with a specially designed instructional insert appropriate for use in low-literacy and non-English-speaking populations, and (3) a reminder postcard. Clinics used their own staff to access tools that had been developed collaboratively by clinic administrators, researchers, and the EHR provider and were embedded in the EHR. Staff used these tools to print materials and assemble mailings periodically (typically monthly or quarterly, but the timing of the mailings was determined by the clinics). Research staff provided additional implementation support by facilitating Plan-Do-Study-Act cycles carried out by staff at each health center.

### Main measures

#### Outcome

The primary outcome for this analysis was completion of a FIT, as identified through EHR laboratory data, after becoming eligible for the intervention. As noted elsewhere, we defined the follow-up interval for outcome assessment for each individual as the earlier of 12 months post initial accrual or August 2015, when intervention activities were initiated in the control clinics [25, 27]. Follow-up windows therefore ranged from 6 to 12 months but were comparably distributed for intervention and control participants.

All participants in the lagged dataset were included in this analysis; those with no evidence of having a returned FIT in their medical record were counted as not completing a FIT. We did not attempt to identify or remove patients who had moved away from the area or had moved their care to another health system.

#### Moderators

We primarily explored moderators related to socioeconomic status, healthcare access, language (as a proxy for recency of immigration), and demographics such as race/ethnicity, which are known to be associated with screening rate disparities. In addition, we explored individual characteristics, identified from the EHR and related administrative data, including age, gender, race, Hispanic ancestry, primary language, federal poverty level category, insurance status, body mass index (BMI), smoking status, whether the participant had a flu shot in the year prior to randomization, whether the participant was current on Pap test and mammography screening (females only), number of Charlson comorbidities [28], and whether they had a visit for diabetes, depression, or a chronic pulmonary condition in the year prior to their enrollment date. The last values entered in the EHR prior to each person’s enrollment date were used for employment status, poverty level, insurance status, and BMI. Neighborhood characteristics were defined based on the participant’s address at the time of enrollment, which was linked via geocoding to variables from the American Community Survey census data [29] and the Centers for Medicare & Medicaid (CMS) Geographic Variation database [30]. These characteristics included emergency department (ED) visits per 1000 CMS enrollees, Generalized Gini Inequality Index [31], median household income, percentage of college graduates, population density, percentage of residents who are at or below the poverty level, and unemployment rate. These neighborhood-level variables were dichotomized based on associated figures in the USA, as close to the year 2014 as possible (for consistency with the timeline of the study). Exact cut-points are shown in Table 1. Dichotomized outcomes were used to enhance interpretability of the findings, although sensitivity analyses were also conducted using the original, continuous measures to ensure dichotomizing the outcomes did not substantially affect the results.

**Table 1:** Cut-points for dichotomized neighborhood-level moderators

Moderator	Description and cut-point for dichotomizing
Gini Inequality	The Gini Index, or index of income concentration, is a statistical measure of income inequality ranging from 0 to 1. A measure of 1 indicates perfect inequality, i.e., one household having all the income and rest having none. A measure of 0 indicates perfect equality, i.e., all households having an equal share of income [31]. Dichotomized at 0.4106, the World Bank estimate of Gini for the USA in 2013 [32]
Unemployment rate	Number of unemployed people as a percentage of the civilian labor force. Dichotomized at 6.6, the US seasonally adjusted unemployment rate in January 2014 [33]
Percentage college graduates	Percentage with bachelor’s degree or higher. Dichotomized at 41.0, the percentage of US citizens aged 55–64 with tertiary education, 2014 [34]
Population density	Total population divided by the land area measured in square miles. Dichotomized at 1000 people/square mile, the definition of a rural tract
Median household income	50th percentile household income for the census tract. Dichotomized at 68,426, the median household income in the USA in 2014, for family households [35]
Poverty (%)	Percentage in census tract living in poverty. Census Bureau uses a set of money income thresholds that vary by family size and composition to determine who is in poverty. If the total income for a family or unrelated individual falls below the relevant poverty threshold, then the family (and every individual in it) or unrelated individual is considered in poverty. Dichotomized at 17.6%, a median split in our study sample
ED visits per 1000 enrollees	Number of emergency department visits per 1000 Medicaid/Medicare enrollees. Dichotomized at 416, the US average in 2013 [36]

*Abbreviations*: *ED* Emergency department

### Statistical analysis

#### Primary analysis

The analytic methods used here are a direct extension of the primary outcome analysis used in the main outcomes paper [25], with the addition of the relevant subgroup variable main effect and treatment interaction terms to permit subgroup-specific treatment estimates and formal estimates of subgroup by treatment interaction. In addition, we used a Poisson rather than logistic link function for the generalized estimating equation (GEE) models and weighted all patients equally to reflect our focus on patient rather than clinic-level effects for this analysis. Finally, we summarized the treatment effects as risk ratios (RRs) rather than as absolute differences or odds ratios for improved comparison with other trials and ease of interpretability. The GEE models used robust variance estimators and specified clinic as a clustering variable to account for intra-clinic correlation. The analysis was conducted in 2017 and 2018.

## Results

We included 30,667 individuals from 26 clinics who were aged 50–74 and were not current on CRC screening. The intervention and usual care groups showed very similar distributions on baseline characteristics, generally within one to four percentage points of each other (Table 2).

**Table 2:** Baseline individual-level patient characteristics

	Allocation				All (*n* = 30,667)	
	Usual care (*n* = 14,904)		Intervention (*n* = 15,763)			
	*N*	%	*N*	%	*N*	%
Age						
50–64	12,249	82.2	12,749	80.9	24,998	81.5
65–75	2655	17.8	3014	19.1	5669	18.5
Gender						
Female	8381	56.2	8605	54.6	16,986	55.4
Male	6523	43.8	7158	45.4	13,681	44.6
Race						
Asian	545	3.8	950	6.4	1495	5.1
Black	629	4.4	743	5.0	1372	4.7
Hawaiian/Pacific Islander	72	0.5	59	0.4	131	0.4
Native American	142	1.0	146	1.0	288	1.0
Other	9	0.1	25	0.2	34	0.1
White	12,886	90.2	13,010	87.1	25,896	88.6
Ethnicity						
Non-Hispanic	12,227	84.6	13,370	88.2	25,597	86.4
Hispanic	2225	15.4	1789	11.8	4014	13.6
Language						
English	12,032	81.6	12,600	81.6	24,632	81.6
Spanish	1797	12.2	1361	8.8	3158	10.5
Other	920	6.2	1475	9.6	2395	7.9
Insurance status						
Uninsured	3494	23.8	3248	20.8	6742	22.3
Medicaid	5642	38.5	6004	38.5	11,646	38.5
Medicare	3562	24.3	3868	24.8	7430	24.5
Commercial	1885	12.8	2392	15.3	4277	14.1
Other	88	0.6	101	0.6	189	0.6
Federal poverty level						
< 100%	5870	48.8	6282	53.8	12,152	51.2
100–150%	2594	21.6	2529	21.7	5123	21.6
151–200%	1258	10.4	1126	9.6	2384	10.0
200%+	2316	19.2	1738	14.9	4054	17.1
Flu shot in 12 months prior to index date						
No	11,144	74.8	11,970	75.9	23,114	75.4
Yes	3760	25.2	3793	24.1	7553	24.6
Mammogram in 2 years prior to index date (women, *n* = 16,986)						
No	5765	68.8	5976	69.4	11,741	69.1
Yes	2616	31.2	2629	30.6	5245	30.9
Pap in 3 years prior to index date (women under age 65, *n* = 13,634)						
No	4085	60.3	4268	62.3	8353	61.3
Yes	2694	39.7	2587	37.7	5281	38.7
Tobacco use						
Current	3892	30.0	4237	30.4	8129	30.2
Former	3335	25.7	3690	26.5	7025	26.1
Never	5768	44.4	6018	43.2	11,786	43.8
BMI						
< 18.5 (underweight)	190	1.4	203	1.4	393	1.4
18.5–25 (normal weight)	3232	23.0	3541	24.5	6773	23.8
25–30 (overweight)	4456	31.7	4431	30.7	8887	31.2
≥ 30 (obese)	6164	43.9	6265	43.4	12,429	43.6
Charlson score, based on past 12 months						
0	7968	53.5	8648	54.9	16,616	54.2
1	4095	27.5	4208	26.7	8303	27.1
2	1633	11.0	1637	10.4	3270	10.7
3+	1208	8.1	1270	8.1	2478	8.1
Visit for chronic pulmonary condition, past 12 months						
No	11,972	80.3	12,716	80.7	24,688	80.5
Yes	2932	19.7	3047	19.3	5979	19.5
Visit for diabetes, past 12 months						
No	11,590	77.8	12,430	78.9	24,020	78.3
Yes	3314	22.2	3333	21.1	6647	21.7
Visit for depression, past 12 months						
No	11,080	74.7	12,083	77.0	23,163	75.9
Yes	3749	25.3	3606	23.0	7355	24.1
Neighborhood ED visits per 1000 Medicaid/Medicare population						
> 419 visits	13,088	88.4	15,167	96.9	28,255	92.8
≤ 419 visits	1722	11.6	485	3.1	2207	7.2
Neighborhood Gini inequality score						
> 0.4106	8197	56.9	9206	60.0	17,403	58.5
≤ 0.4106	6216	43.1	6146	40.0	12,362	41.5
Neighborhood median household income						
≤ $68,426	13,114	91.0	14,654	95.4	27,768	93.3
> $68,426	1299	9.0	698	4.6	1997	6.7
Neighborhood percentage college graduates						
≤ 41%	11,537	80.0	12,854	83.7	24,391	81.9
> 41%	2876	20.0	2499	16.3	5375	18.1
Neighborhood population density per square mile						
≤ 1000 (rural)	4423	30.7	5907	38.5	10,330	34.7
> 1000 (non-rural)	9991	69.3	9446	61.5	19,437	65.3
Neighborhood poverty (percentage below 100% FPL)						
> 17.6%	7384	51.2	8281	53.9	15,665	52.6
≤ 17.6%	7029	48.8	7071	46.1	14,100	47.4
Neighborhood unemployment rate						
> 6.6%	12,691	88.0	13,978	91.0	26,669	89.6
≤ 6.6%	1722	12.0	1375	9.0	3097	10.4

*Abbreviations*: *ED* Emergency department, *FPL* Federal poverty level

Most of the persons in the sample were aged 50–64 (81.5%), White (88.6%), and non-Hispanic (86.4%), and more than half were female (55.4%). Most of the participants had household incomes that were below 200% of the federal poverty level (82.3%), and the most common form of health coverage was Medicaid (38.5%), followed by Medicare (24.5%), and no insurance coverage (22.3%). Records suggested relatively low completion preventive services; 24.6% had a flu shot in the past year, 30.9% of women had a mammogram in the past 2 years, and 38.7% of age-eligible women had a recent Pap smear.

Tables 3 and 4 show the percentage of patients who had completed a FIT in the subgroups of interest, by intervention group. Although not always statistically significant, we saw a consistent pattern of increased FIT return rates among intervention participants compared to control participants across all subgroups studied, with incidence rate ratios (IRRs) generally ranging from 1.25 to 1.50. FIT completion in the intervention group ranged from 15 and 25% for most subgroups, typically three to six percentage points higher than the control group participants. Also shown in Tables 3 and 4 are the relative risks for having completed a FIT (vs. not) in each subgroup and the *P* value for the treatment*moderator interaction. The only moderator with a statistically significant interaction was race; persons of Asian descent showed a twofold response to the intervention (adjusted incident rate ratio [aIRR] = 2.06, 95% confidence interval [CI] 1.41 to 3.00). Intervention response was in the more typical range for participants who were White (aIRR = 1.32, 95% CI 0.99 to 1.76) and Black (aIRR = 1.28, 95% CI 0.85 to 1.92). Among persons of Asian descent, 18.9% in the usual care group completed a FIT, compared with 37.7% in the intervention group. In contrast, usual care completion rates among White and Black persons were 12.9 and 14.9%, respectively, compared to 15.8 and 20.2% for the intervention group participants.

**Table 3:** FIT completion by individual-level patient characteristics

Subgroup variable	Number of participants	Percentage completed FIT	Percentage completed FIT		Adjusted IRR^a^ (95% CI)	Interaction *P* value
			UC	IG		
Age						
50–64	24,998	15.2	13.0	17.3	1.36 (1.02, 1.81)	0.58
65–74	5669	16.9	14.4	19.0	1.41 (1.03, 1.94)	
Gender						
Female	16,986	16.3	14.2	18.3	1.33 (1.00, 1.78)	0.33
Male	13,681	14.5	12.0	16.8	1.42 (1.05, 1.90)	
Race						
White	25,896	14.4	12.9	15.8	1.32 (0.99, 1.76)	0.003
Black	1372	17.8	14.9	20.2	1.28 (0.85, 1.92)	
Asian	1495	30.8	18.9	37.7	2.06 (1.41, 3.01)	
Hispanic ancestry						
Non-Hispanic	25,597	14.8	12.5	16.8	1.39 (1.05, 1.85)	0.19
Hispanic	4014	21.2	18.7	24.3	1.25 (0.91, 1.71)	
Primary language						
English	24,632	13.5	11.8	15.1	1.38 (1.04, 1.84)	0.89
Non-English	5553	25.5	20.2	30.6	1.40 (1.03, 1.90)	
Insurance status						
Uninsured	6742	16.0	13.3	19.1	1.29 (0.94, 1.78)	0.67
Medicaid	11,646	16.2	13.3	18.9	1.38 (1.02, 1.88)	
Medicare	7430	15.5	13.8	17.1	1.34 (0.98, 1.84)	
Commercial	4227	13.0	12.8	13.3	1.48 (1.05, 2.08)	
Federal poverty level						
≤ 100%	12,152	16.8	14.4	19.0	1.29 (0.95, 1.75)	0.42
> 100–150%	5123	16.1	13.3	18.9	1.37 (0.99, 1.89)	
> 151–200%	2384	14.9	13.0	17.1	1.27 (0.88, 1.83)	
> =200%	4054	14.4	11.9	17.7	1.51 (1.07, 2.11)	
Flu shot past year						
No	23,114	14.2	12.1	16.2	1.39 (1.04, 1.86)	0.46
Yes	7553	19.4	16.7	22.2	1.32 (0.98, 1.79)	
Mammogram in past 2 years						
No	11,741	14.7	12.7	16.5	1.27 (0.96, 1.67)	0.18
Yes	5245	20.0	17.5	22.5	1.42 (1.06, 1.90)	
Pap test in last 3 years						
No	8353	14.2	12.4	16.0	1.28 (0.97, 1.70)	0.40
Yes	5281	19.0	16.6	21.5	1.37 (1.03, 1.84)	
Smoking status						
Former or never	18,811	17.3	14.6	19.9	1.40 (1.04, 1.87)	0.16
Current	8129	12.3	10.8	13.7	1.25 (0.91, 1.72)	
Body mass index						
< 30.0 kg/m^2^	16,053	15.8	13.0	18.5	1.44 (1.07, 1.93)	0.08
≥ 30.0 kg/m^2^	12,429	15.7	13.9	17.4	1.28 (0.95, 1.72)	
Charlson comorbidities						
0–2	28,189	15.7	13.5	17.8	1.35 (1.01, 1.80)	0.16
≥ 3	2478	13.2	10.4	15.9	1.61 (1.11, 2.32)	
Visits for chronic pulmonary disease						
No	24,688	15.7	13.4	17.8	1.35 (1.01, 1.80)	0.50
Yes	5979	14.7	12.3	17.0	1.42 (1.04, 1.95)	
Visits for diabetes						
No	24,020	14.8	12.4	17.0	1.42 (1.06, 1.89)	0.06
Yes	6647	18.1	16.3	20.0	1.23 (0.91, 1.67)	
Visits for depression						
No	23,163	16.1	13.7	18.3	1.37 (1.03, 1.84)	0.66
Yes	7355	13.9	12.1	15.8	1.33 (0.98, 1.81)	

*Abbreviations*: *FIT* Fecal immunochemical test, *IG* Intervention group, *IRR* Incidence rate ratio, *UC* Usual care group, *CI* Confidence interval

^a^Data based on mixed effects Poisson regression analysis controlling for clinic level clustering and adjusting for subgroup variable and, as applicable, age, gender, and health center

**Table 4:** FIT completion by patients’ neighborhood characteristics

Subgroup variable	Number of participants	Percentage completed FIT	Percentage completed FIT		Adjusted IRR^a^ (95% CI)	Interaction *P* value
			UC	IG		
ED visits per 1000 Medicaid/Medicare population						
> 419 visits	28,255	16.1	14.2	17.8	1.34 (1.00, 1.78)	0.36
≤ 419 visits	2207	8.5	6.8	14.6	1.73 (0.95, 3.14)	
Gini Inequality score						
> 0.4106	17,403	15.2	13.1	17.1	1.37 (1.02, 1.83)	0.81
≤ 0.4106	12,362	16.1	13.5	18.8	1.39 (1.04, 1.86)	
Median household income						
≤ $68,426	27,768	15.6	13.2	17.7	1.37 (1.03, 1.82)	0.67
> $68,426	1997	15.4	14.1	17.8	1.44 (0.99, 2.10)	
Percentage college graduates						
≤ 41%	24,391	15.7	13.2	17.9	1.38 (1.04, 1.84)	0.61
> 41%	5375	15.1	13.6	16.8	1.33 (0.96, 1.83)	
Population density per square mile						
≤ 1000 (rural)	10,330	13.4	10.6	15.5	1.45 (1.07, 1.96)	0.26
> 1000 (non-rural)	19,437	16.7	14.5	19.2	1.32 (0.99, 1.78)	
Poverty (percentage below 100% FPL)						
> 17.6%	15,665	16.2	13.8	18.5	1.30 (0.97, 1.74)	0.06
≤ 17.6%	14,100	14.8	12.8	16.9	1.47 (1.10, 1.98)	
Unemployment rate						
> 6.6%	26,669	15.4	13.1	17.4	1.37 (1.03, 1.82)	0.50
≤ 6.6%	3097	17.4	14.6	20.9	1.46 (1.04, 2.04)	

*Abbreviations*: *CI* Confidence interval, *ED* Emergency department, *FIT* Fecal immunochemical test, *FPL* Federal poverty level, *Gini* The Gini Index, or index of neighborhood income concentration, higher number means greater inequality, range 0–1.0, *IG* Intervention group, *IRR* Incidence rate ratio, *UC* Usual care group

^a^Data based on mixed effects Poisson regression analysis controlling for clinic level clustering and adjusting for subgroup variable and, as applicable, age, gender, and health center

Although no other interaction tests were statistically significant, a few other characteristics were statistically significant at *P* = 0.10. Specifically, we found larger effects for those with non-obese range BMIs than for participants with BMI ≥ 30.0 (aIRR = 1.44 vs. 1.28, *P* = 0.08), for those without vs. with a visit for diabetes in the past year (aIRR = 1.42 vs. 1.23, *P* = 0.06), and those living in lower poverty vs. higher poverty neighborhoods (aIRR = 1.47 vs. 1.30, *P* = 0.06). However, the preponderance of evidence suggests that intervention effects were fairly consistent across patient subpopulations. We reran the analyses of BMI and the neighborhood-level characteristics that we dichotomized, keeping the moderators as continuous variables (data not shown). None of the interaction terms were statistically significant in these analyses (*P* > 0.14 in all cases), supporting the robustness of these findings.

## Discussion

In this population, drawn from safety net clinics in Oregon, Washington, and California serving low-income patients, a wide range of patient subpopulations generally showed fairly comparable responses to the mailed FIT intervention. However, the intervention effect was largest among persons of Asian descent, with a statistically significant incident rate ratio of 2.06 (95% CI 1.41 to 3.00). It is unclear why this subgroup showed large effects, and this result needs replication. One possible explanation we explored was that 77% of persons of Asian descent in the study population reported that English was not their preferred language, so it was possible that the wordless FIT instructions developed for this trial were particularly helpful for the Asian subpopulation. However, we did not find a greater benefit of the intervention among non-English speakers in general, nor was there a parallel effect in persons of Hispanic descent, who had a similar proportion of non-English speakers (76%) as the Asian subpopulation.

We found two other trials of mailed FIT interventions that reported on moderators of treatment effect [14, 16], although these trials did not report specifically on differential effects in persons of Asian descent compared to other race/ethnic groups. One of these mailed FIT trials found that the intervention effect was comparable across age, gender, race/ethnicity (Hispanic vs. other), preferred language (English vs. Spanish), and insurance status, but did find a larger treatment effect among persons with no visits during the follow-up period than those with three or more visits, a variable we did not explore [14]. In their study, among persons with no visits during follow-up, 3 % of the control group participants and 59% of the intervention group participants had completed a FIT within 6 months, a 56 percentage point difference between groups. Among those with three or more visits during follow-up, 58% of the control group and 86% of the intervention group completed a FIT within 6 months, a 28 percentage point difference. The other trial of mailed FITs that reported effect moderators found no differences in intervention response by gender or race/ethnicity (comparing non-Hispanic white, black, and Hispanic subgroups) [16].

We adhered to most recommendations outlined by the checklist for the appraisal of moderators and predictors (CHAMP) [37]. First, we examined characteristics related to those that have been shown to be related to CRC screening rates, including demographics, socioeconomic factors, health status, and use of preventive services. The broad factors were selected a priori; however, the specific fields were restricted to those available in the EHR and in the databases of neighborhood-level data use by this study. We used measures taken prior to the start of the interventions, employed statistical interaction testing, and presented results for all moderators examined. In addition, the setting and study population were comparable to the settings and populations in which the mail FIT would be used clinically. Because of the large number of moderators we examined, the relatively small number of participants of Asian descent, and the lack of an effect related to non-English language preference (a construct related to Asian ethnicity), we view the finding of a positive moderating effect in Asian patients as exploratory and in need of replication. We also believe the overall pattern of consistent benefit across a range of patient characteristics in this setting is plausible.

One of the main limitations of this study is related to our reliance on the EHR for capture of moderator variables. Patients in this low-income population may be more mobile than typical, both in terms of where they live and where they receive their healthcare. As such, the neighborhood-level characteristics may not be current for people who struggle with homelessness or insecure housing, and healthcare-related services may be received at non-study clinics. However, low-income patients’ mobility is likely primarily between neighborhoods with similar economic profiles, so we believe the information on neighborhood-level characteristics will often remain reasonably similar when patients have moved. However, EHRs are simply not always complete and accurate, so some patients will have been dropped from some analyses due to missing moderator data and some will have been misclassified in the EHR. In addition, some participants may have completed a FIT within a health system that was not covered by the OCHIN collaborative, so they would be misclassified as not completing a FIT.

Another limitation of our study is that we tested a larger number of potential moderators without adjusting our analyses to maintain a type I error rate of 5%. Thus, even though we did find one statistically significant interaction indicating a larger benefit for patients of Asian descent, this finding may be due to chance and not be robust to replication. An additional limitation is that we did not conduct power calculations specifically for the moderator analyses, given the wide range of subgroup sizes, and analyses for some subgroups may be underpowered.

Despite these limitations, our study has a number of important strengths. Our sample included more than 30,000 patients, and so had substantial numbers of patients across a variety of patient subgroups. In addition, these clinics are part of a collaborative that uses a common EHR system, meaning differences in data storage and capture were minimized across the clinics and that data on study participants seen at other clinics under the umbrella EHR provider would be captured. Another very important strength is that this was a pragmatic effectiveness trial, conducted in real-world safety net clinics, using the existing staff and infrastructure. While the overall effect of this intervention was not as large as that seen in some other trials of mailed FITs, the effect was robust across patient subpopulations and was implemented within the constraints of real-world, low-resourced clinics.

The relatively modest effects of an automated FIT mailing intervention were generally consistent across a wide range of patient subpopulations, suggesting broad impact that is unlikely to exaggerate existing disparities in CRC screening rates. Patients of Asian descent may be more likely to benefit from the intervention; however, this finding needs to be replicated.

## Conclusions

Response to a mailed FIT intervention was generally consistent across a wide range of individual and neighborhood-level patient characteristics, including typically underserved patients and those in low-resource communities.

## Data Availability

The deidentified data used during the current study are available from the corresponding author on reasonable request.

## Abbreviations

aIRR: Adjusted incidence rate ratio
BMI: Body mass index
CMS: Centers for Medicare & Medicaid Services
CRC: Colorectal cancer
EHR: Electronic health record
FIT: Fecal immunochemical test
GEE: Generalized estimating equation
HMO: Health maintenance organization
IRR: Incidence rate ratio
RR: Risk ratio
STOP CRC: Strategies and Opportunities to Stop Colon Cancer in Priority Populations
USPSTF: United States Preventive Services Task Force
